# Supplementary material for: A chicken DNA methylation clock for the prediction of broiler health
Source: Commun Biol. 2021 Jan 18;4:76. doi: 10.1038/s42003-020-01608-7 (PMC7814119; doi:10.1038/s42003-020-01608-7)
Supplement: Supplementary file 2 — Supplementary Information [file 42003_2020_1608_MOESM2_ESM.pdf]

Supplementary Material

A chicken DNA methylation clock for the prediction of broiler health

Günter Raddatz, Ryan J. Arsenault, Bridget Aylward, Rose Whelan, Florian Böhl, and Frank Lyko

Contents:

Supplementary Tables S1-S6

Supplementary Figures S1-S2

Tab. S1. Published whole-genome bisulfite sequencing datasets analyzed in this study.

**accession no.**	**tissue**	**strain**	**no. of samples**	**coverage (combined)**	**conversion**
GSE56975	lung	Fayoumi/Leghorn	4	57.6x	94.3%
PRJNA352686	breast	Gushi	2	19.6x	99.7%
GSE56639	sperm	unknown	1	24.8x	99.7%

Conversion indicates the bisulfite conversion efficiency.

Tab. S2. The 20 most significantly enriched transcription factor binding motifs in LMRs.

Motif name	p-value	q-value	On target (%)	Background (%)	Enrichment
CTCF	1e-1510	0	6.87	1.03	6.67
BORIS	1e-864	0	6.87	1.81	3.80
ERG	1e-414	0	21.68	14.21	1.53
Etv2	1e-386	0	13.42	7.74	1.73
Fli1	1e-345	0	14.74	9.06	1.63
ETV1	1e-339	0	17.17	11.07	1.55
ETS1	1e-335	0	14.21	8.69	1.64
GABPA	1e-248	0	10.85	6.65	1.63
Gata2	1e-230	0	9.28	5.54	1.68
Gata1	1e-224	0	8.43	4.93	1.71
Gata4	1e-207	0	12.91	8.66	1.49
EHF	1e-205	0	15.59	10.93	1.43
GATA3	1e-181	0	17.26	12.64	1.37
Ets1-distal	1e-171	0	4.25	2.14	1.99
PU.1	1e-122	0	5.86	3.65	1.60
DMRT6	1e-109	0	2.33	1.1	2.12
DMRT1	1e-106	0	2.67	1.35	1.98
Elk4	1e-105	0	5.31	3.34	1.59
SPDEF	1e-104	0	11.95	8.95	1.34
ELF5	1e-104	0	8.38	5.88	1.43

Tab. S3. Comparison of the animal methylation clock training datasets.

**organism**	**# of samples**	**clock span**	**tissues**	**method**	**reference**
Broiler chicken	36	35 days	spleen, breast muscle, ileum, jejunum	WGBS	this study
Mouse	62	41 weeks	liver, lung, heart, brain	RRBS	[1]
Dog	46	14 years	blood	RRBS	[2]
Wolf	62	14 years	blood	RRBS	[2]
Naked mole rat	24	22 years	liver	BS-PCR	[3]
Humpback whale	45	30 years	skin	BS-PCR	[4]
Sea brass	50	10 years	muscle	BS-PCR	[5]

Methods: WGBS – whole-genome bisulfite sequencing, RRBS – reduced representation bisulfite sequencing, BS-PCR – bisulfite PCR.

Table S4. Clock LMRs (alpha = 0.9, lambda = 0.3147).

ID	chrom	start	end	weight	ileum	spleen	breast	jejunum
1	chr1	3310966	3311076	5.106	0.089	0.117	0.048	0.108
2	chr1	13486724	13487721	-1.078	0.421	0.180	0.224	0.424
3	chr1	77403928	77404268	5.291	0.106	0.160	0.040	0.183
4	chr1	131728204	131729184	-6.235	0.407	0.363	0.318	0.197
5	chr1	135369614	135369882	-1.194	0.436	0.184	0.403	0.419
6	chr1	165806748	165806816	-0.009	0.477	0.527	0.844	0.542
7	chr2	31315302	31315823	0.961	0.148	0.099	0.104	0.200
8	chr2	31316250	31316368	15.824	0.129	0.087	0.059	0.111
9	chr2	91174537	91175128	-26.554	0.235	0.262	0.188	0.238
10	chr4	1489570	1490794	-8.003	0.176	0.149	0.158	0.214
11	chr4	8453114	8454528	3.325	0.159	0.524	0.316	0.211
12	chr4	31342294	31342536	0.228	0.638	0.574	0.638	0.640
13	chr5	839708	840094	2.227	0.231	0.328	0.153	0.233
14	chr5	1942052	1942842	2.613	0.325	0.414	0.204	0.349
15	chr5	39059304	39059368	0.307	0.025	0.068	0.024	0.058
16	chr5	52951604	52951808	2.676	0.070	0.148	0.024	0.091
17	chr6	8416236	8416588	12.930	0.130	0.200	0.099	0.160
18	chr8	13056204	13056776	4.557	0.142	0.269	0.122	0.150
19	chr9	23812486	23812678	6.756	0.155	0.382	0.179	0.151
20	chr11	675295	675546	-3.678	0.316	0.329	0.638	0.346
21	chr12	9433040	9433568	9.905	0.406	0.351	0.132	0.409
22	chr12	16248172	16248357	-0.539	0.598	0.583	0.815	0.317
23	chr13	13146980	13147888	-10.892	0.167	0.113	0.135	0.179
24	chr13	16716156	16716440	-0.540	0.153	0.273	0.166	0.180
25	chr14	4137806	4137912	-6.589	0.259	0.137	0.232	0.215
26	chr15	8945390	8945554	-3.262	0.493	0.464	0.741	0.324
27	chr18	2358384	2359684	-2.706	0.448	0.368	0.364	0.472
28	chr19	9052179	9052244	-9.309	0.601	0.295	0.258	0.523
29	chr20	11718627	11718916	20.167	0.149	0.379	0.193	0.201
30	chr23	5568088	5568140	-2.259	0.402	0.290	0.436	0.439
31	chr25	1101298	1101396	-0.093	0.493	0.267	0.204	0.416
32	chr26	4608324	4608370	2.441	0.163	0.416	0.228	0.203
Intercept of linear model equation found by glmnet: 17.345								

Correction factors are indicated for different tissues. For correction, the corresponding value has to be subtracted.

Table S5. Clock CpGs (alpha = 0.7, lambda = 0.4016).

ID	chrom	position	weight	ileum	spleen	breast	jejunum
1	chr1	26806096	-0.333	0.636	0.475	0.464	0.64
2	chr1	27051068	-1.207	0.363	0.124	0.445	0.235
3	chr1	79412910	-3.879	0.467	0.438	0.573	0.414
4	chr1	193007724	-0.894	0.504	0.181	0.398	0.44
5	chr2	84879641	2.595	0.381	0.665	0.191	0.415
6	chr2	139780944	-0.004	0.32	0.198	0.053	0.182
7	chr3	9654592	-2.179	0.503	0.328	0.698	0.589
8	chr3	23119819	-2.285	0.282	0.251	0.31	0.292
9	chr3	32240754	2.209	0.256	0.244	0.148	0.264
10	chr3	55893779	-3.285	0.528	0.563	0.673	0.564
11	chr3	55933564	-0.301	0.335	0.302	0.649	0.165
12	chr4	20608622	-0.825	0.547	0.512	0.554	0.728
13	chr4	48345505	0.468	0.285	0.435	0.239	0.304
14	chr4	70292571	-0.001	0.254	0.235	0.561	0.332
15	chr5	1942965	3.015	0.268	0.532	0.178	0.322
16	chr5	1942982	2.248	0.334	0.562	0.174	0.397
17	chr5	12844701	-0.238	0.583	0.435	0.711	0.691
18	chr5	16850281	1.412	0.651	0.784	0.654	0.723
19	chr5	17507391	-3.468	0.261	0.197	0.115	0.351
20	chr5	39037892	1.739	0.476	0.506	0.379	0.61
21	chr5	54227250	-1.625	0.225	0.358	0.361	0.28
22	chr5	58662889	5.718	0.46	0.621	0.364	0.503
23	chr6	5240214	-0.287	0.262	0.317	0.196	0.213
24	chr6	7819244	4.26	0.209	0.511	0.234	0.188
25	chr6	12024016	-2.447	0.662	0.24	0.575	0.515
26	chr6	12065954	1.12	0.286	0.388	0.249	0.325
27	chr7	9815074	-5.1	0.726	0.46	0.738	0.655
28	chr7	11137846	-0.002	0.367	0.286	0.587	0.326
29	chr7	14040077	-1.945	0.431	0.309	0.357	0.366
30	chr7	21995171	-2.653	0.192	0.057	0.244	0.137
31	chr7	30586853	0.837	0.335	0.391	0.176	0.501
32	chr8	3444574	1.024	0.255	0.654	0.388	0.256
33	chr8	8196471	0.618	0.56	0.802	0.691	0.565
34	chr8	18912606	-1.112	0.442	0.333	0.599	0.542
35	chr8	27250408	-0.755	0.473	0.413	0.394	0.735
36	chr10	20035839	-0.002	0.251	0.14	0.142	0.234
37	chr11	7627454	0.396	0.593	0.601	0.222	0.672
38	chr14	9143159	-3.085	0.519	0.34	0.564	0.355
39	chr14	9143204	-2.843	0.678	0.401	0.615	0.388
40	chr15	201524	6.892	0.596	0.634	0.3	0.559
41	chr15	8945553	-13.223	0.766	0.724	0.87	0.542
42	chr17	1673086	-0.441	0.616	0.305	0.472	0.669
43	chr19	7327224	5.149	0.657	0.492	0.266	0.648
44	chr23	172291	-0.279	0.646	0.538	0.562	0.479
45	chr23	5568087	-1.692	0.277	0.183	0.18	0.255
Intercept of linear model equation found by glmnet: 17.365							

Correction factors are indicated for different tissues. For correction, the corresponding value has to be subtracted.

Table S6. Inflammatory phenotypes, as determined by kinome peptide analysis.

Jejunum day 15 post-hatch			
**GO term ID**	**Term description**	**Observed protein count**	**FDR**
GO:0002376	immune system process	137	9.54E-36
GO:0002682	regulation of immune system process	103	9.16E-34
GO:0002684	positive regulation of immune system process	83	2.85E-33
GO:0050776	regulation of immune response	82	8.68E-33
GO:0002764	immune response-regulating signaling pathway	56	1.95E-31
GO:0050778	positive regulation of immune response	67	7.94E-31
GO:0002768	immune response-regulating cell surface receptor signaling pathway	47	1.39E-28
GO:0002757	immune response-activating signal transduction	50	1.25E-27
GO:0002253	activation of immune response	52	2.07E-26
GO:0002429	immune response-activating cell surface receptor signaling pathway	40	8.54E-24

Jejunum day 16 post-hatch			
**GO term ID**	**Term description**	**Observed protein count**	**FDR**
GO:0002376	immune system process	120	8.83E-33
GO:0050776	regulation of immune response	73	1.34E-30
GO:0002684	positive regulation of immune system process	71	9.63E-29
GO:0002682	regulation of immune system process	87	2.09E-28
GO:0002764	immune response-regulating signaling pathway	49	3.28E-28
GO:0050778	positive regulation of immune response	59	4.45E-28
GO:0002757	immune response-activating signal transduction	45	5.57E-26
GO:0002253	activation of immune response	47	4.55E-25
GO:0045088	regulation of innate immune response	42	5.95E-22
GO:0006955	immune response	82	6.18E-22

Jejunum day 35 post-hatch			
**GO term ID**	**Term description**	**Observed protein count**	**FDR**
GO:0002764	immune response-regulating signaling pathway	50	6.2E-34
GO:0002682	regulation of immune system process	80	5.61E-31
GO:0050776	regulation of immune response	65	2.02E-30
GO:0002757	immune response-activating signal transduction	44	3.1E-29
GO:0002684	positive regulation of immune system process	62	1.16E-27
GO:0002253	activation of immune response	45	1.55E-27
GO:0002376	immune system process	97	1.85E-27
GO:0045088	regulation of innate immune response	43	6.8E-27
GO:0050778	positive regulation of immune response	50	2.41E-25
GO:0002768	immune response-regulating cell surface receptor signaling pathway	37	3.81E-25

Observed protein counts represent statistically significantly differentially phosphorylated proteins in CpG injected broiler chickens compared to control GpC injected chickens.

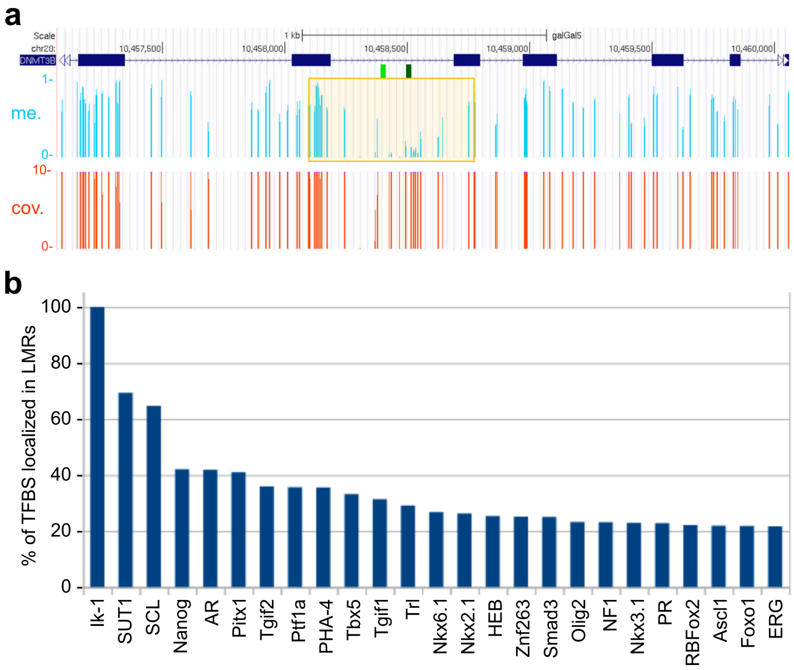

Fig. S1. Association of chicken LMRs with transcription factor binding sites. (a) Representative LMR (shaded orange) with binding sites for transcription factors Ik-1 (light green box) and CTCF (dark green box) indicated und the gene model. The methylation track shows DNA methylation levels (light blue) and sequencing coverage levels (red) for an LMR from the chicken DNMT3B locus (chromosome 20) in lung tissue. Sequencing coverages were cut off at >10. (b) Barplot showing the fraction of the top 25 transcription factor binding sites localized in LMRs.

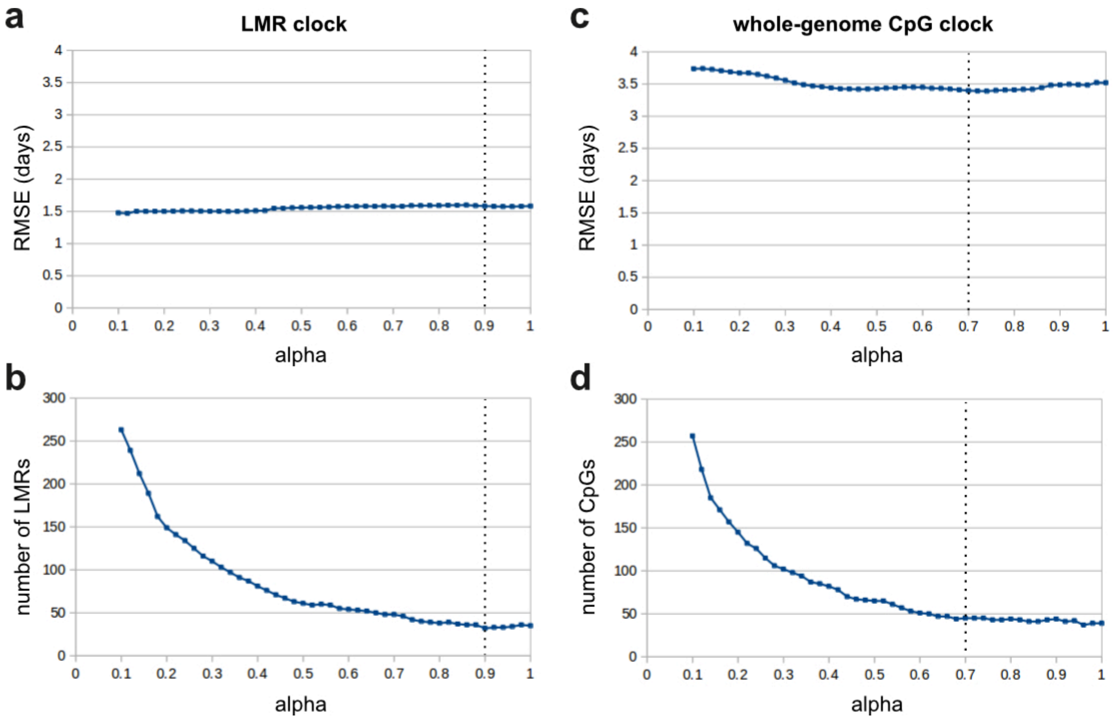

Fig. S2. Performance of the training process with 6-fold cross-validation depending on the parameter alpha for normalized data. (a) RMSE for LMR clocks. (b) Number of CpGs in LMR clocks. (c) RMSE for whole-genome CpG clocks. (d) Number of CpGs in whole-genome CpG clocks. Dashed lines mark the chosen values of alpha.

Supplementary References

1. Stubbs, T. M. *et al.* Multi-tissue DNA methylation age predictor in mouse. *Genome Biol.* **18**, 68 (2017).
2. Thompson, M. J., VonHoldt, B., Horvath, S. & Pellegrini, M. An epigenetic aging clock for dogs and wolves. *Aging (Albany. NY).* **9**, 1055–1068 (2017).
3. Lowe, R. *et al.* DNA methylation clocks as a predictor for ageing and age estimation in naked mole-rats, Heterocephalus glaber. *Aging (Albany. NY).* **12**, 4394–4406 (2020).
4. Polanowski, A. M., Robbins, J., Chandler, D. & Jarman, S. N. Epigenetic estimation of age in humpback whales. *Mol. Ecol. Resour.* **14**, 976–987 (2014).
5. Anastasiadi, D. & Piferrer, F. A clockwork fish: Age prediction using DNA methylation-based biomarkers in the European seabass. *Mol. Ecol. Resour.* **20**, 387–397 (2020).
